# Cognition impairment and risk of subclinical cardiovascular disease in older adults: The atherosclerosis risk in communities study

**DOI:** 10.3389/fnagi.2022.889543

**Published:** 2022-07-27

**Authors:** Dongze Li, Yu Jia, Jing Yu, Yi Liu, Fanghui Li, Wei Zhang, Yongli Gao, Xiaoyang Liao, Zhi Wan, Zhi Zeng, Rui Zeng

**Affiliations:** ^1^Department of Emergency Medicine and West China School of Nursing, Disaster Medicine Center, West China Hospital, Sichuan University, Chengdu, China; ^2^Department of Cardiology, West China Hospital, Sichuan University, Chengdu, China; ^3^Department of General Practice and National Clinical Research Center for Geriatrics, West China Hospital, Sichuan University, Chengdu, China

**Keywords:** cognitive function, cardiovascular disease, high-sensitivity cardiac troponin T, N-terminal pro-B-type natriuretic peptide, Atherosclerosis Risk in Communities Study (ARIC)

## Abstract

**Background:**

Clinical cardiovascular disease (CVD) and cognition impairment are common and often coexist in aging populations, and CVD is associated with greater cognition impairment risk; however, the association between cognition impairment and CVD risk is inconsistent. It is unknown if pathways that contribute to CVD are caused by impaired cognition. We hypothesized that cognition impairment would be associated with greater subclinical CVD including subclinical myocardial damage [assessed by high-sensitivity cardiac troponin T (hs-cTnT)] and cardiac strain or dysfunction [assessed by N-terminal pro-B-type natriuretic peptide (NT-proBNP)].

**Methods:**

This analysis included Atherosclerosis Risk in Communities Study (ARIC) participants who underwent global cognition *z*-score tests between 1991 and 1993. Cardiac biomarkers were measured from stored plasma samples collected between 1996 and 1999. Logistic regression models were used to determine the association of cognitive function with subclinical CVD risk.

**Results:**

There were 558/9216 (6.1%) and 447/9097 (5.0%) participants with incident elevated hs-CTnT (≥14 ng/L) and NT-proBNP (≥300 pg/mL) levels, respectively. Comparing the lowest and highest quartiles of global cognition *z*-scores, a higher incidence of elevated hs-CTnT [odds ratio (OR) = 1.511, 95% confidence interval (CI): 1.093–2.088, *P* = 0.013] and NT-proBNP (OR = 1.929, 95% CI: 1.350–2.755, *P* < 0.001) were observed, respectively. In structural equation modeling, the indirect effect of global cognition *z*-score on major adverse cardiac events was 42.1% (*P* < 0.05).

**Conclusion:**

Impairments in baseline cognitive function were associated with subclinical myocardial damage or wall strain. Although future studies are warranted to investigate the pathophysiological mechanisms behind these associations, our study suggests common pathways between cognitive and cardiac dysfunction.

## Introduction

Cardiovascular disease (CVD) is the leading cause of premature mortality worldwide (Barquera et al., 2015). Furthermore, disability caused by CVD remains extremely high, and thus, CVD is also the leading cause of disability-adjusted life years (DALYs), which are expected to rise to 150 million by 2020 (Bansilal et al., 2015). Knowledge about the risk factors of subclinical CVD is critical to optimize prevention strategies and may help to clarify the mechanism connecting these risk factors to CVD (Kuller et al., 1995; Newman et al., 1999; O’Leary et al., 1999).

Sufficient evidence has confirmed that cognitive impairment, as an early and sensitive indicator of injury of cerebral vessels and parenchyma, is a common complication of CVD (Ampadu and Morley, 2015; Scheltens et al., 2016; Deckers et al., 2017; Fujiyoshi et al., 2018; Rensma et al., 2020). Although not all studies have shown cognitive impairment as an independent risk factor for CVD (Skoog et al., 2005; Singh-Manoux et al., 2009), most studies suggest that individuals with impaired cognition have higher risk of stroke, cardiovascular events, and mortality (Ferrucci et al., 1996; de Moraes et al., 2003; Elkins et al., 2005; O’Donnell et al., 2012; Bagai et al., 2019). This association may be explained by several possible mechanisms. First, CVD risk factors are common in covert stroke and Alzheimer’s disease (Fillit et al., 2008; Alonso et al., 2009), thus cognitive impairment may simply be a marker of a high CVD risk population. Second, cognitive impairment may increase CVD risk by leading to multiple unhealthy lifestyles (Zhao et al., 2021), such as an unhealthy diet, sedentary activities, alcoholism, etc. Last but not least, stroke and Alzheimer’s disease has an important influence on the renin-angiotensin system (Nakagawa and Sigmund, 2017), autonomic nervous system (Dorrance and Fink, 2015), and mental health (Kales et al., 2005; Monastero et al., 2009), all of which were validated risk factors of CVD (Rosengren et al., 2004; Miller and Arnold, 2019). Whatever, confirming the relationship between cognitive impairment and subclinical CVD can further prove that there is a bidirectional relationship between cognitive impairment and CVD. However, almost no study has investigated the association between cognitive impairments and subclinical CVD. It is unknown if pathways that contribute to CVD, such as subclinical myocardial injury or cardiac wall strain or dysfunction, are partly caused by impaired cognition.

Considerable evidence demonstrated that cardiac troponin (cTn) is the preferred biomarker for the assessment of myocardial injury (Thygesen et al., 2012b; Apple et al., 2015), and an elevated cTn value above the 99th percentile URL is defined as myocardial injury according to the standard of the Fourth Universal Definition of Myocardial Infarction (2018) (Thygesen et al., 2018). In addition, high-sensitivity (hs)-cTn assays are recommended for routine clinical use since they have higher diagnostic sensitivity and accuracy for myocardial injury than cTn (Thygesen et al., 2012a,b). The elevated N-terminal pro-B type natriuretic peptide (NT-proBNP) concentration is an effective indicator of increased wall stress and volume expansion; thus, NT-proBNP is a powerful predictive marker for major adverse cardiac events (MACE). Therefore, hs-cTnT and NT-proBNP are highly sensitive indicators of subclinical CVD that represent two distinct pathways by which CVD may be caused by myocardial injury (hs-cTnT) or cardiac strain or dysfunction (NT-proBNP) (Yancy et al., 2013; Afilalo et al., 2014; Mueller et al., 2019). In this study, we aimed to investigate whether baseline cognitive function is associated with increased hs-cTnT or NT-proBNP in individuals without known cardiovascular disease.

## Materials and methods

### Study population

The Atherosclerosis Risk in Communities Study (ARIC) study began in 1987, and four field centers in ARIC (Washington County, Maryland, Forsyth County, North Carolina, Jackson City, Missouri, and Minneapolis) were randomly selected and recruited approximately 4,000 people between the ages of 45 and 64 years from specific populations in their communities as cohort samples (No authors listed, 1989). A total of 15,792 participants were comprehensively examined, including medical, social, and demographic data. The participants were re-examined every 3 years, and the first screening (baseline) was conducted in 1987–1989. Participants were asked to respond to phone calls every six months and attend ARIC exam visits periodically. The study program was approved by the institutional review committees of all study sites, and all participants provided written informed consent.

**FIGURE 1:**
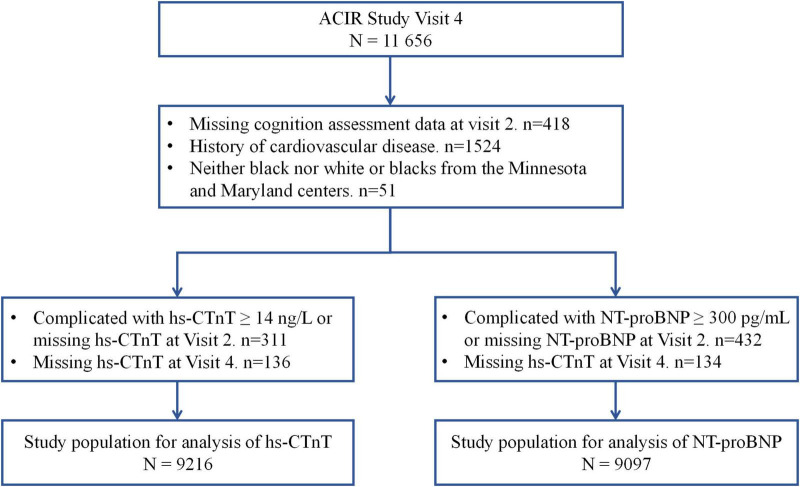
Study flow chart. hs-CTnT, high-sensitive cardiac troponin T; NT-proBNP, N-terminal pro-B-type natriuretic peptide.

In this study, we used Visit 2 (1991–1993) as our baseline. In Visit 4, we excluded individuals without cognition assessment data at Visit 2 (*n* = 418), prevalent CVD (*n* = 1,524), and participants from the Minnesota and Maryland centers (*n* = 51). In the hs-CTnT group, we excluded individuals whose hs-CTnT levels were ≥14 ng/L or were missing hs-CTnT data at Visit 2 (*n* = 311) or Visit 4 (*n* = 136). Finally, 9,216 participants were included in the hs-CTnT group (Figure 1). For the NT-proBNP group, we excluded individuals whose NT-proBNP levels were ≥300 pg/mL or were missing NT-proBNP data at Visit 2 (*n* = 432) or Visit 4 (*n* = 134). Thus, 9,097 participants were included in the NT-proBNP group (Figure 1).

### High-sensitivity cardiac troponin T and N-terminal pro-B-type natriuretic peptide

High-sensitivity cardiac troponin T was measured using a sandwich immunoassay on the Roche Elecsys 2010 Analyzer (Roche Diagnostics, Indianapolis, IN, United States) from plasma samples collected at Visit 2 and stored at −70°C. The detection limit of hs-cTnT was 6 ng/L and the limit of blank was 3 ng/L. For individuals with undetectable hs-cTnT levels, a value of 1.5 ng/L was imputed. The interassay coefficient of variation for hs-cTnT was 6.4% (at a mean control of 29 ng/L) (Matsushita et al., 2018). An elevated hs-cTnT level was set as ≥14 ng/L, according to the 99th percentile value reported by the manufacturer, and defined as subclinical myocardial damage (Thygesen et al., 2018). NT-proBNP was also measured from plasma samples on the Roche Elecsys 2010 Analyzer. The lower limit of detection was 5 pg/mL. For individuals with undetectable NT-proBNP levels, a value of 2.5 ng/L was imputed. The interassay coefficient of variation for NT-proBNP was 7.4% (at a mean control of 134 pg/mL) (Nambi et al., 2013). An elevated NT-proBNP level was set as ≥300 ng/L according to previous pre-specified levels (Blaha et al., 2016; Lazo et al., 2016; Hussain et al., 2021), yielding a 98% negative predictive value to exclude heart failure (Januzzi et al., 2006). The predictive values of these cut-off points for CVD were also validated by previous ARIC studies (Saunders et al., 2011; Hussain et al., 2021).

### Cognitive assessment

In Visits 2, 4, and 5, professional researchers conducted three cognitive tests on each participant in a standard order in a quiet room. These tests included a delayed word recall test (DWRT), digit symbol substitution test (DSST), and word fluency test (WFT), which measured memory, executive function, and language function, respectively (Kim et al., 2017; Li et al., 2020). In the DWRT, participants needed to learn a word and apply the word to one or two sentences. After 5 min, participants had 1 min to recall ten words, and their score was the correct number of words (Knopman and Ryberg, 1989). In the DSST, participants used a keyboard to translate symbols into numbers, and their score was the number of correct translations within 90 s (Jaeger, 2018). In the WFT, participants were asked to generate words starting with the letters F, A, and S at 1-min intervals, excluding proper nouns, and their score was the number of words generated (Pendleton et al., 1982). The global cognition *z*-score was calculated as an average of the three individual *z*-scores at each study visit and standardized using the global *z*-score mean and standard deviation (SD) of Visit 2. In addition, one of the magnetic resonance imaging (MRI) sub-studies, five other neurocognitive tests were performed: logical memory test, incidental learning, animal naming score, and trail making tests A and B. In the factor analysis, the ARIC cohort was treated as a single group, and the global cognition, language domain, memory domain, and executive functioning domain factor scores were obtained.

### Major adverse cardiac events

Major adverse cardiac events were defined as a definite or probable stroke, myocardial infarction, or definite coronary heart disease (CHD) death that occurred after cognitive testing.

### Covariate assessment

Baseline demographic, lifestyle, and clinical characteristics were obtained in Visit 2. Education was divided into basic education (less than high school), secondary education (high school), and higher education (college). Self-reported income was divided into high (>$35,000), middle ($16,000–35,000), and low (<$16,000) incomes. Self-reported drinking was divided into current, former, and never. Self-reported smoking was divided into current, former, and never smokers. Body mass index was defined as weight in kilograms divided by the height squared. The sitting blood pressure was measured twice with a sphygmomanometer, and the average value of the two measurements was recorded. Hypertension was defined as self-reported antihypertensive drug use or blood pressure ≥140/90 mmHg. Diabetes was defined as fasting blood glucose ≥126 mg/dL, non-fasting blood glucose ≥200 mg/dL, or self-reported antidiabetic drugs. Fasting blood glucose was collected at baseline and measured using the modified hexokinase or glucose-6-phosphate dehydrogenase method. History of CVD was defined as myocardial infarction (self-reported or diagnosed by electrocardiographic changes), revascularization, or hospitalization for heart failure at or before Visit 4. The definition of chronic obstructive pulmonary disorder (COPD) was based on the doctor’s self-reported diagnosis or obstructive vital capacity measurement model, which was defined as the ratio of forced expiratory volume in one second (FEV1)/FVC <0.70. Total cholesterol was determined using an enzymatic method, and high-density lipoprotein cholesterol levels were measured after dextran-magnesium precipitation of non-high-density lipoprotein particles.

### Statistical analysis

The participants were categorized into quartiles (Q1, Q2, Q3, and Q4) based on the global cognition *z*-score and factor score. Parametric and nonparametric continuous variables are reported as the mean ± SD and median (25th and 75th percentiles) and compared using analysis of variance and Mann–Whitney U test, respectively. Categorical variables are reported as frequencies and percentages, and compared using a Chi-squared test.

For the prospective analyses, the main outcomes were increased hs-CTnT (≥14 ng/L) and NT-proBNP (≥300 pg/mL) levels at Visit 4. The logistic regression model was used to assess the relationship between the categorical baseline cognitive function and incidence of elevated hs-CTnT and NT-proBNP. To further determine whether these relationships were independent risk factors, the model was adjusted for age, sex, center-race, education (<high school, high school, or >high school), annual household income (<$16,000, $16,000–25,000, $25,000–35,000, $35,000–50,000, or >$50,000), smoking (never, former, and current), drinking (never, former, and current), body mass index, systolic blood pressure, heart rate, total cholesterol, triglycerides, high-density lipoprotein, hypertension, diabetes, and chronic obstructive pulmonary disease. In addition, subgroup analyses were stratified by age (<54 versus ≥54 years), sex, race (black versus white), hypertension (yes versus no), and diabetes mellitus (yes versus no).

To explore the indirect effect of subclinical CVD determined by the global cognition *z*-score on MACE, path analysis was established by structural equation modeling (Stein et al., 2017). The results of the path analysis were calculated using standardized regression coefficients (β) to show the direct and indirect effects on MACE.

A two-tailed *P* < 0.05 was considered significant for all tests. All statistical analyses were performed using SPSS version 26.0 (IBM Corp, Armonk, NY, United States), and R software 3.5.0 (Vienna, Austria).

### Data availability

ARIC data are available through NIH NHLBI-sponsored Biologic Specimen and Data Repository Information Coordinating Center (BioLINCC) at https://biolincc.nhlbi.nih.gov.

## Results

### Baseline characteristics

Among the 9,216 and 9,097 subjects included in our prospective, there were 558 (6.1%) and 447 (5.0%) participants with incident elevated hs-CTnT (≥14 ng/L) and NT-proBNP (≥300 pg/mL), respectively, at Visit 4. Baseline (1991–1993) characteristics are described and compared in Table 1. Compared to the population with lower cardiac biomarkers, participants with elevated hs-CTnT (≥14 ng/L) and NT-proBNP (≥300 pg/mL) were older, more likely to be female, African American, and smoker, complicated with hypertension and diabetes, have lower education, and higher concentrations of cardiovascular risk markers in midlife (*P* < 0.05 for all comparisons). Similarly, patients with lower global cognition *z*-scores had more cardiovascular risk factors described above (*P* < 0.05 for all, Supplementary Table 1).

**TABLE 1:** Baseline (1991–1993) participant characteristics by classified cardiac biomarkers at Visit 4 (1996–1999).

Characteristic	hs-CTnT (*N* = 9,216)			NT-proBNP (*N* = 9,097)		
		
	<14 ng/L (*N* = 8,658)	≥14 ng/L (*N* = 558)	*P*	<300 pg/mL ng/L (*N* = 8,650)	≥300 pg/mL (*N* = 447)	*P*
**Demographic variables**						
Age, years	56.5 ± 5.6	59.4 ± 5.8	<0.001	56.5 ± 5.6	59.9 ± 5.5	<0.001
Male sex	3,435/8,658 (39.7)	148/558 (26.7)	<0.001	3,682/8,650 (42.6)	158/447 (35.3)	0.003
African Americans	1,713/8,658 (19.8)	137/558 (24.6)	0.006	1,787/8,650 (20.7)	72/447 (16.1)	0.020
Education			<0.001			0.002
Less than high school	1,446/8,647 (16.7)	138/557 (24.8)		1,454/8,639 (16.8)	103/445 (23.1)	
High school	2,815/8,647 (32.6)	135/557 (24.2)		2,765/8,639 (32.0)	138/445 (31.0)	
College	4,386/8,647 (50.7)	284/557 (51.0)		4,420/8,639 (51.2)	204/445 (45.8)	
Income, US$			0.017			<0.001
<16,000	1,321/8,210 (16.1)	103/526 (19.6)		1,084/7,978 (13.6)	80/404 (19.8)	
16,000–35,000	4,500/8,210 (54.8)	296/526 (56.3)		4,496/7,978 (56.4)	229/404 (56.7)	
>35,000	2,389/8,210 (29.1)	127/526 (24.1)		2,398/7,978 (30.1)	95/404 (23.5)	
Smoking			<0.001			<0.001
Never	3,786/8,653 (43.8)	197/558 (35.3)		3,785/8,646 (43.8)	160/447 (35.8)	
Former	3,215/8,653 (37.2)	252/558 (45.2)		3,255/8,646 (37.6)	175/447 (39.1)	
Current	1,652/8,653 (19.1)	109/558 (19.5)		1,606/8,646 (18.6)	112/447 (25.1)	
Drinking			0.001			0.556
Never	1,945/8,653 (22.5)	110/557 (19.7)		1,937/8,645 (22.4)	92/447 (20.6)	
Former	1,534/8,653 (17.7)	133/557 (23.9)		1,571/8,645 (18.2)	88/447 (19.7)	
Current	5,174/8,653 (59.8)	314/557 (56.4)		5,137/8,645 (59.4)	267/447 (59.7)	
**Physiological and lab variables**						
Body mass index, kg/m^2^	27.6 ± 5.2	29 ± 5.2	<0.001	27.8 ± 5.2	27.8 ± 5.5	0.994
SBP, mmHg	119.2 ± 17.0	128.6 ± 20.9	<0.001	119.3 ± 17.1	128.1 ± 21.5	<0.001
DBP, mmHg	71.6 ± 9.8	74.1 ± 11.1	<0.001	71.8 ± 9.8	72.8 ± 11.2	0.039
Heart rate, /min	65.3 ± 9.7	66.1 ± 13.2	0.039	65.4 ± 9.8	64.9 ± 9.9	0.339
Total cholesterol, mg/dl	5.4 ± 1.0	5.3 ± 1.0	0.007	5.4 ± 1.0	5.4 ± 1.0	0.598
HDL, mg/dl	1.3 ± 0.4	1.1 ± 0.4	<0.001	1.3 ± 0.4	1.3 ± 0.5	0.050
LDL, mg/dl	3.4 ± 0.9	3.4 ± 0.9	0.740	3.4 ± 0.9	3.4 ± 0.9	0.620
Triglycerides, mg/dl	1.5 ± 0.9	1.7 ± 1.2	<0.001	1.5 ± 0.9	1.5 ± 0.8	0.810
Creatinine, mg/mL	1.1 ± 0.2	1.3 ± 0.9	<0.001	1.1 ± 0.2	1.2 ± 0.3	<0.001
Blood glucose, mmol/L	108.5 ± 31.7	126.4 ± 53.9	<0.001	6.1 ± 1.9	6.4 ± 2.7	<0.001
**Chronic medical conditions**						
Hypertension	2,099/8,638 (24.3)	235/557 (42.2)	<0.001	2,109/8,630 (24.4)	187/447 (41.8)	<0.001
Diabetes mellitus	883/8631 (10.2)	144/552 (26.1)	<0.001	972/8,619 (11.3)	70/445 (15.7)	0.004
COPD	1,653/8,531 (19.4)	158/547 (28.9)	<0.001	1,650/8,526 (19.4)	121/438 (27.6)	<0.001
**Cognition score**						
Global cognition *z*-score	0.2 ± 0.9	−0.3 ± 1.0	<0.001	0.3 ± 2.2	−0.4 ± 2.2	<0.001
DWRT score	6.8 ± 1.4	6.2 ± 1.6	<0.001	6.8 ± 1.5	6.6 ± 1.6	0.007
DSST score	47.1 ± 13.3	41.3 ± 14.6	<0.001	46.8 ± 13.5	41.3 ± 11.6	<0.001
WFT score	34.5 ± 12.2	31.7 ± 12.4	<0.001	34.4 ± 12.3	33.2 ± 12.2	0.053
Global cognition factor score	0.2 ± 0.8	−0.2 ± 0.9	<0.001	0.1 ± 0.8	−0.1 ± 0.8	<0.001
Language domain factor score	0.1 ± 0.6	−0.1 ± 0.7	<0.001	0.1 ± 0.7	0 ± 0.7	0.119
Memory domain factor score	0.1 ± 0.4	−0.1 ± 0.4	<0.001	0 ± 0.4	0 ± 0.5	0.024
Executive functioning domain factor score	0.1 ± 0.5	−0.1 ± 0.5	<0.001	0.1 ± 0.5	0 ± 0.5	<0.001

Values are expressed as n/N (%), mean ± SD, and median (25th and 75th). Global cognition z-score was calculated by computing the mean from the z-score versions of the DSST, WFT, and DWRT administered during the Visit 2.

SBP, systolic blood pressure; DBP, diastolic blood pressure; HDL, high density lipoprotein; LDL, Low density lipoprotein; COPD, chronic obstructive pulmonary disease; DSST, digit symbol substitution test; WFT, word fluency test; DWRT, delayed word recall test.

Additionally, participants with elevated hs-CTnT (≥14 ng/L) had significant lower global cognition *z*-scores; DSST, WFT, and DWRT scores; global cognition factor score; and language, memory, and executive functioning domain factor scores (*P* < 0.001 for all comparisons). As for participants with elevated NT-proBNP (≥300 pg/mL), they only had significant lower global cognition *z*-scores, DSST score, global cognition factor score, and executive functioning domains factor score (*P* < 0.001 for all comparisons).

### Cognitive function and cardiac biomarker

For individual tests of cognitive function, risk-factor adjusted odds ratio (OR) for elevated hs-CTnT (≥14 ng/L) and NT-proBNP (≥300 pg/mL) levels are presented in Table 2. Comparing Q1 and Q4 of global cognition *z*-scores and factor scores, a higher incidence of elevated hs-CTnT [OR = 1.511, 95% confidence interval (CI): 1.093–2.088, *P* = 0.013; OR = 1.564, 95% CI: 1.123–2.177, *P* = 0.008, respectively) was observed. Impaired cognitive function was also associated with NT-proBNP levels ≥300 pg/mL (global cognition *z*-score: OR = 1.929, 95% CI: 1.350–2.755, *P* < 0.001; global cognition factor score: OR = 1.506, 95% CI: 1.050–2.158, *P* = 0.026) but not with NT-proBNP levels ≥100 pg/mL (data not shown). Complete risk-factor-adjusted logistic models for each cognitive test are presented in Supplementary Table 2. Significant associations of DWRT, DSST, and memory and executive functioning domain factor scores with elevated hs-CTnT were observed. Significant associations of DSST score and executive functioning domain factor score with elevated NT-proBNP were observed.

**TABLE 2:** Adjusted ORs (95% CI) for the association of baseline (1991–1993) cognition function with incident elevated hs-cTnT and NT-proBNP.

Variable	Quartile	Incident elevated hs-CTnT (≥14 ng/L)		Incident elevated NT-proBNP (≥300 pg/mL)	
			
		OR (95% CI)	*P*-trend	OR (95% CI)	*P*-trend
Global cognition *z*-score			0.062		0.003
	Q1	1.511 (1.093–2.088)	0.013	1.929 (1.350–2.755)	<0.001
	Q2	1.184 (0.868–1.613)	0.286	1.300 (0.932–1.813)	0.122
	Q3	1.136 (0.830–1.555)	0.425	1.384 (1.007–1.902)	0.046
	Q4	Reference	–	Reference	–
Global cognition factor score			0.001		0.048
	Q1	1.564 (1.123–2.177)	0.008	1.506 (1.050–2.158)	0.026
	Q2	0.947 (0.692–1.296)	0.734	1.010 (0.731–1.396)	0.951
	Q3	0.997 (0.731–1.361)	0.987	1.047 (0.768–1.427)	0.771
	Q4	Reference	–	Reference	–

Multivariate logistic regression analysis between cognition function and incident elevated cardiac biomarkers adjusted by age, sex, center-race, education (<high school, high school, or >high school), annual household income (<16,000, 16,000–25,000, 25,000–35,000, 35,000–50,000, or >50,000), smoking (never, former, and current), drinking (never, former, and current), body mass index, systolic blood pressure, heart rate, total cholesterol, triglycerides, high density lipoprotein, hypertension, diabetes, and chronic obstructive pulmonary disease. hs-CTnT, high-sensitive cardiac troponin T; NT-proBNP, N-terminal pro-B-type natriuretic peptide; OR, odds ratio; CI, confidence interval.

### Subgroup analysis

We found similar results of risk-factor adjusted ORs when we stratified individuals by age (<54 versus ≥54 years), sex, hypertension (yes versus no), and diabetes mellitus (yes versus no). There were no significant interactions between these risk factors, cognition scores, and elevated cardiac biomarkers (Tables 3, 4). Furthermore, impaired cognitive function was not associated with elevated cardiac biomarkers for African Americans, and a significant interaction between race, cognition scores, and elevated cardiac biomarkers was observed (*P* < 0.05).

**TABLE 3:** Adjusted ORs (95% CIs) for the association of baseline (1991–1993) cognition function with incident elevated hs-cTnT in different subgroups.

Subgroup	Adjusted OR (95% CI) for incident elevated hs-CTnT (≥14 ng/L)				*P* for interaction
	
	Q1	Q2	Q3	Q4	
**Global cognition *z*-score**					
Age*					0.200
<54 year	**1.496 (1.056–2.613)**	1.269 (0.769–2.093)	0.873 (0.511–1.491)	Reference	
≥54 year	**1.272 (1.084–1.912)**	1.034 (0.694–1.543)	1.149 (0.772–1.711)	Reference	
Gender					0.068
Female	**1.900 (1.054–3.425)**	0.904 (0.508–1.611)	1.105 (0.647–1.888)	Reference	
Male	**1.557 (1.062–2.283)**	1.415 (0.981–2.039)	1.255 (0.861–1.831)	Reference	
Race					0.011
White	**1.522 (1.079–2.146)**	1.261 (0.919–1.731)	1.202 (0.877–1.646)	Reference	
Black	7.251 (0.961–54.714)	4.799 (0.622–37.057)	3.963 (0.482–32.564)	Reference	
Hypertension					0.168
No	**1.587 (1.055–2.390)**	1.353 (0.928–1.971)	1.266 (0.867–1.850)	Reference	
Yes	**1.620 (1.161–2.730)**	1.086 (0.646–1.826)	1.100 (0.648–1.867)	Reference	
Diabetes					0.455
No	**1.456 (1.012–2.096)**	1.183 (0.841–1.664)	1.281 (0.917–1.790)	Reference	
Yes	1.696 (0.988–3.519)	1.240 (0.602–2.554)	0.721 (0.320–1.625)	Reference	
**Global cognition factor score**					
Age*					0.645
<54 year	**1.378 (1.030–2.466)**	0.849 (0.501–1.438)	0.911 (0.551–1.506)	Reference	
≥54 year	**1.341 (1.084–2.035)**	0.835 (0.560–1.246)	0.929 (0.620–1.392)	Reference	
Gender					0.123
Female	**1.676 (1.181–2.379)**	1.116 (0.812–1.533)	1.051 (0.770–1.435)	Reference	
Male	2.176 (0.493–9.616)	0.911 (0.191–4.350)	0.681 (0.108–4.275)	Reference	
Race					0.007
White	**2.083 (1.147–3.784)**	0.881 (0.497–1.562)	0.975 (0.569–1.672)	Reference	
Black	**1.576 (1.069–2.325)**	1.078 (0.746–1.558)	1.014 (0.697–1.476)	Reference	
Hypertension					0.398
No	**1.828 (1.215–2.751)**	1.116 (0.766–1.626)	0.948 (0.646–1.391)	Reference	
Yes	**1.542 (1.189–2.646)**	0.855 (0.502–1.456)	1.117 (0.666–1.875)	Reference	
Diabetes					0.136
No	**1.477 (1.023–2.133)**	0.963 (0.683–1.357)	1.066 (0.764–1.486)	Reference	
Yes	**1.967 (1.091–4.211)**	1.012 (0.487–2.100)	0.640 (0.281–1.460)	Reference	

*Population were divided by median of variables. Bold value indicates P < 0.05. Multivariate logistic regression analysis between cognition function and incident elevated cardiac biomarkers adjusted by age, sex, center-race, education (<high school, high school, or >high school), annual household income (<16,000, 16,000–25,000, 25,000–35,000, 35,000–50,000, or >50,000), smoking (never, former, and current), drinking (never, former, and current), body mass index, systolic blood pressure, heart rate, total cholesterol, triglycerides, high density lipoprotein, hypertension, diabetes, and chronic obstructive pulmonary disease.

hs-CTnT, high-sensitive cardiac troponin T; NT-proBNP, N-terminal pro-B-type natriuretic peptide; OR, odds ratio; CI, confidence interval.

**TABLE 4:** Adjusted ORs (95% CIs) for the association of baseline (1991–1993) cognition function with incident elevated NT-proBNP in different subgroups.

Subgroup	Adjusted OR (95% CI) for incident elevated NT-proBNP (≥300 pg/mL)				*P* for interaction
	
	Q1	Q2	Q3	Q4	
**Global cognition *z*-score**					
Age*					0.099
<54 year	**1.372 (1.070–2.660)**	1.027 (0.580–1.819)	1.253 (0.752–2.086)	Reference	
≥54 year	**2.205 (1.421–3.423)**	**1.450 (0.951–2.212)**	**1.495 (0.988–2.262)**	Reference	
Gender					0.322
Female	**2.287 (1.479–3.535)**	1.096 (0.730–1.646)	**1.457 (1.014–2.094)**	Reference	
Male	**1.803 (1.192–3.509)**	**1.933 (1.022–3.655)**	1.328 (0.677–2.607)	Reference	
Race					0.006
White	**1.985 (1.366–2.885)**	**1.345 (0.955–1.893)**	**1.360 (0.982–1.882)**	Reference	
Black	2.686 (0.336–21.46)	1.589 (0.186–13.557)	2.838 (0.331–24.348)	Reference	
Hypertension					0.127
No	**1.567 (1.017–2.461)**	1.155 (0.772–1.727)	1.259 (0.862–1.839)	Reference	
Yes	**2.847 (1.527–5.308)**	1.762 (0.957–3.246)	1.791 (0.985–3.254)	Reference	
Diabetes					0.547
No	**2.056 (1.401–3.018)**	1.189 (0.829–1.705)	**1.485 (1.065–2.070)**	Reference	
Yes	**1.856 (1.316–4.104)**	**1.906 (1.020–5.044)**	0.718 (0.229–2.250)	Reference	
**Global cognition factor score**					
Age*					0.579
<54 year	0.967 (0.475–1.967)	0.878 (0.495–1.555)	0.972 (0.586–1.610)	Reference	
≥54 year	**1.710 (1.109–2.635)**	1.056 (0.705–1.580)	1.086 (0.729–1.618)	Reference	
Gender					0.091
Female	**1.660 (1.058–2.602)**	0.981 (0.663–1.452)	1.194 (0.839–1.699)	Reference	
Male	**1.373 (1.072–2.615)**	1.094 (0.596–2.007)	0.760 (0.396–1.460)	Reference	
Race					0.008
White	**1.623 (1.113–2.368)**	1.041 (0.745–1.455)	1.048 (0.764–1.438)	Reference	
Black	1.515 (0.184–12.458)	1.309 (0.154–11.124)	1.703 (0.181–16.007)	Reference	
Hypertension					0.639
No	1.276 (0.980–2.017)	0.901 (0.605–1.341)	0.955 (0.657–1.388)	Reference	
Yes	**2.144 (1.156–3.974)**	1.300 (0.731–2.314)	1.340 (0.759–2.365)	Reference	
Diabetes					0.085
No	**1.595 (1.085–2.346)**	0.992 (0.702–1.402)	1.068 (0.771–1.479)	Reference	
Yes	1.168 (0.389–3.506)	1.133 (0.416–3.090)	0.952 (0.332–2.731)	Reference	

*Population were divided by median of variables. Bold value indicates P < 0.05. Multivariate logistic regression analysis between cognition function and incident elevated cardiac biomarkers adjusted by age, sex, center-race, education (<high school, high school, or >high school), annual household income (<16,000, 16,000–25,000, 25,000–35,000, 35,000–50,000, or >50,000), smoking (never, former, and current), drinking (never, former, and current), body mass index, systolic blood pressure, heart rate, total cholesterol, triglycerides, high density lipoprotein, hypertension, diabetes, and chronic obstructive pulmonary disease.

hs-CTnT, high-sensitive cardiac troponin T; NT-proBNP, N-terminal pro-B-type natriuretic peptide; OR, odds ratio; CI, confidence interval.

### Path analysis

**FIGURE 2:**
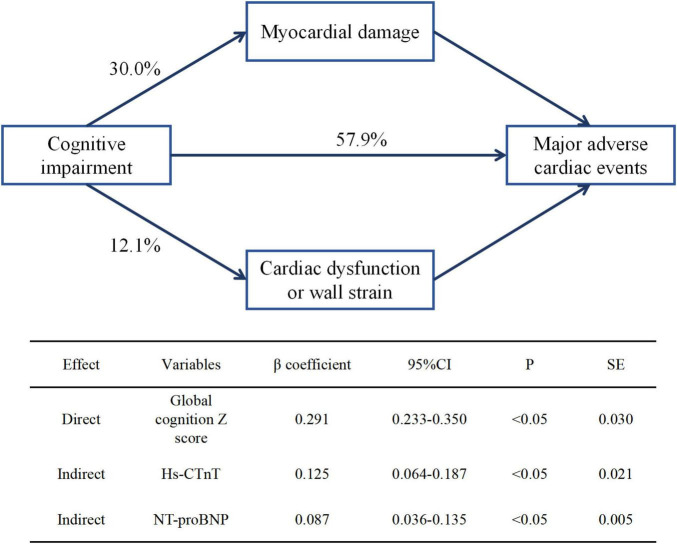
Direct and indirect effects of global cognition *z*-score on subclinical cardiovascular disease and long-term major adverse cardiac events. β coefficient was calculated by standard regression equation. hs-CTnT, high-sensitive cardiac troponin T; NT-proBNP, N-terminal pro-B-type natriuretic peptide.

The global cognition *z*-score was significantly associated with both elevated cardiac biomarkers and long-term MACE by path analysis (*P* < 0.001). In structural equation modeling, the indirect effects of global cognition *z*-scores on myocardial injury (hs-cTnT) and cardiac strain or dysfunction (NT-proBNP) were 30.0% (β coefficient, 0.125) and 12.1% (β coefficient, 0.087), respectively (Figure 2).

## Discussion

In this large cohort of the ARIC study, we found a significant inverse association between baseline cognitive function and subclinical CVD, expressed as elevated hs-CTnT (≥14 ng/L) and NT-proBNP (≥300 pg/mL). The relationship was independent of all other measured prognostic factors. Participants with the lowest cognition score had approximately a 50% higher risk of myocardial injury, while only participants with the lowest executive functioning domain factor score had an increased risk of cardiac strain or dysfunction. In addition, >40% of cognitive impairment effects on MACE were mediated by subclinical CVD. Our findings suggest that cognitive impairments could be important risk factors for subclinical CVD and may shed light on novel, independent pathways linking cognitive function to CVD among older adults without a history of clinical CVD.

**FIGURE 3:**
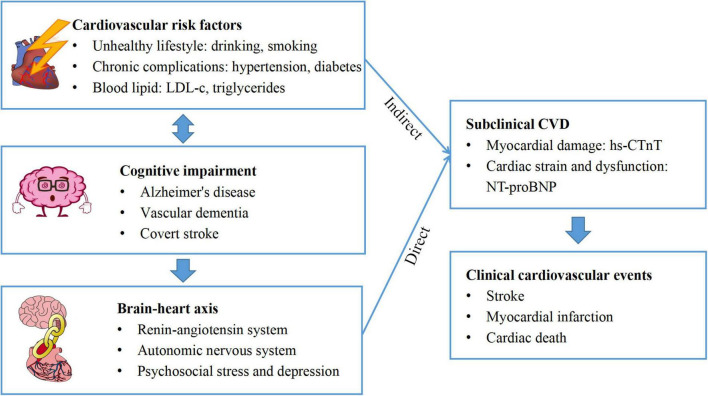
Possible mechanisms of cognitive impairment and subclinical cardiovascular disease (CVD).

An association between cognitive impairment and incidence of elevated hs-CTnT and NT-proBNP would be expected for several reasons. According to our results, individuals with cognitive impairment have significantly more modifiable CVD risk factors, such as unhealthy lifestyle (heavy drinking, smoking, and obesity), chronic complications (hypertension and diabetes), and blood lipid (low-density lipoprotein cholesterol and triglycerides). All of these factors are also risk factors for covert stroke, vascular dementia, and Alzheimer’s disease (Fillit et al., 2008; Alonso et al., 2009), the most common causes of cognitive decline; thus, the relationship between cognitive impairment and subclinical CVD may be indirectly mediated by CVD risk factors (Iqbal et al., 2008; Anstey et al., 2009; Scarmeas et al., 2009). More importantly, according to the theory of the brain-heart axis (Manea et al., 2015; Riching et al., 2020), cognitive impairment is a manifestation of encephalopathy, which leads to abnormal cardiac structure and function *via* activation of the renin-angiotensin system (Nakagawa and Sigmund, 2017; Miller and Arnold, 2019), dysfunction of autonomic nervous system (Dorrance and Fink, 2015; Miller and Arnold, 2019), increased psychosocial stress and affective disorder (Rosengren et al., 2004; Kales et al., 2005; Monastero et al., 2009). In a/the structural equation, >40% of cognitive impairment effects on MACE were mediated by subclinical CVD. Therefore, cognitive impairment may be an indicator of high-risk CVD patients through indirect and direct pathways (Figure 3).

The relationship between cognitive impairment and subclinical CVD was consistent in subgroups of age, sex, hypertension, and diabetes. However, these associations were not significant for African Americans in our study, which may be due to the following reasons: compared with Caucasians, African Americans have lower baseline cognitive level, which may be affected by many other confounding factors, such as education, socioeconomic status, and acculturation (Mungas et al., 2009; Norman et al., 2011; Mascialino et al., 2019). Moreover, African Americans are more likely to have early cardiovascular events (Zhao et al., 2019), however, those participants were excluded from our study, so there may be a selective bias in this study. Future studies need to provide powerful evidence for African Americans.

Findings from prior studies examining the association between cognitive function and CVD have been inconsistent, and have primarily focused on stroke risk alone rather than all individual CV events. Several articles evaluated the association between cognitive impairment and CV events, of which, two reported a non-significant association (Ferrucci et al., 1996; de Moraes et al., 2003; Elkins et al., 2005; Skoog et al., 2005; Singh-Manoux et al., 2009; O’Donnell et al., 2012; Bagai et al., 2019). In addition, previous studies usually analyzed a single cognitive score (such as MMSE) and domain and showed that executive dysfunction is a prominent manifestation of early vascular cognitive impairment, which is the most significant risk factor for CVD and mortality (O’Donnell et al., 2012). This study evaluated the relationship of multiple cognitive assessment tools (DWRT, DSST, and WFT) and cognitive domains (language, memory, and executive function) with subclinical CVD. Our findings suggest that simple cognitive screening tests, used commonly by general practitioners, can help identify patients at an increased risk of subclinical CVD, even after adjusting for a larger number of lifestyle and CV risk factors. Similar to previous reports, executive dysfunction is the only independent risk factor for wall strain and dysfunction. Notably, this study explains the possible relationship between cognitive impairment and CVD from a novel perspective and suggests that myocardial injury and wall strain and dysfunction may be the key pathways of cognitive impairment leading to CVD.

Our study has several limitations. Subclinical CVD can not only be defined by cardiac biomarkers, but also includes cardiac structure abnormalities. The relationship between cognitive function and cardiac structure was not analyzed. The interactions of race and cognitive function with cardiac biomarkers could be stochastic and should be further investigated. Despite multiple imputations, residual bias from selective attrition is possible because missing cardiac biomarkers assessments may not be random, especially for African Americans, and it is difficult to draw clear conclusions. In addition, it is unknown whether the observed association would be consistent using other cognition assessments. Although the results of this study were adjusted for multiple confounding factors, there may still be potential variables that were not included, such as repression and psychological stress. Additional factors influencing the association of cognitive function with these biomarkers, such as analysis type and biological variation in biomarker sampling and changes of biomarkers over time, were not analyzed in this study. Finally, our results are mainly derived from middle-aged people in the community, so the generalizability for younger individuals or individuals outside the community setting is unknown.

## Conclusion

In conclusion, cognitive function impairments were associated with elevations biomarkers of subclinical cardiac damage (CTnT) and/or wall strain (NT-proBNP), which may be contribute toward CVD. Therefore, cognitive impairment is an independent risk factor for subclinical CVD, and targeted interventions to improve cognitive function may reduce the incidence of subclinical and clinical CVD for participants with no known CVD. However, future studies are needed to verify these findings.

## Data availability statement

Publicly available datasets were analyzed in this study. This data can be found here: https://biolincc.nhlbi.nih.gov/.

## Ethics statement

The studies involving human participants were reviewed and approved by the West China Hospital. The patients/participants provided their written informed consent to participate in this study.

## Author contributions

DL, YJ, and RZ designed the research. DL, YJ, and YL analyzed the data under the supervision of XL and ZZ. DL and YJ wrote the first draft of the manuscript. JY, YL, FL, WZ, YG, XL, and ZW reviewed the manuscript and provided critical scientific input. RZ had main responsibility for the final content of the manuscript. All authors approved the final draft of the manuscript.

## Abbreviations

ARIC: Atherosclerosis Risk in Communities Study
CHD: coronary heart disease
CI: confidence interval
COPD: chronic obstructive pulmonary disorder
CVD: cardiovascular disease
DALYs: disability-adjusted life years
DWRT: delayed word recall test
DSST: digit symbol substitution test
FEV1: forced expiratory volume in one second
hs-cTnT: high-sensitivity cardiac troponin T
NT-proBNP: N-terminal pro-B-type natriuretic peptide
MACE: major adverse cardiac events
OR: odds ratio
WFT: word fluency test.
