# Supplementary material for: Cognition impairment and risk of subclinical cardiovascular disease in older adults: The atherosclerosis risk in communities study
Source: Front Aging Neurosci. 2022 Jul 27;14:889543. doi: 10.3389/fnagi.2022.889543 (PMC9363767; doi:10.3389/fnagi.2022.889543)
Supplement: Supplementary file 1 [file Table_1.DOCX]

Supplementary Table 1. Baseline (1991-1993) participant characteristics by quartile of global cognition z-score

| Characteristic | | Global cognition z-score (N=8880) | | | | |
| --- | --- | --- | --- | --- | --- | --- |
|  |  | Q1  (N=2220) | Q2  (N=2219) | Q3  (N=2219) | Q4  (N=2222) | P |
| **Demographic Variables** | |  |  |  |  |  |
|  | Age, years | 58.1 ± 5.69 | 56.9 ± 5.56 | 56.4 ± 5.55 | 55.1 ± 5.33 | <0.001 |
|  | Male sex | 1065 (48.0) | 1217 (54.8) | 1382 (62.3) | 1540 (69.3) | <0.001 |
|  | Americans | 1253 (56.4) | 1823 (82.2) | 1956 (88.1) | 2069 (93.1) | <0.001 |
|  | Education |  |  |  |  | <0.001 |
|  | Less than high school | 940/2216 (42.4) | 345/2215 (15.6) | 160/2217 (7.22) | 50/2220 (2.25) |  |
|  | High school | 649/2216 (29.3) | 812/2215 (36.7) | 761/2217 (34.3) | 619/2220 (27.9) |  |
|  | College | 627/2216 (28.3) | 1058/2215 (47.8) | 1296/2217 (58.5) | 1551/2220 (69.9) |  |
|  | Income, US$ |  |  |  |  |  |
|  | <16 000 | 736/2066 (35.6) | 328/2097 (15.6) | 184/2130 (8.64) | 98/2119 (4.62) | <0.001 |
|  | 16 000-35 000 | 1096/2066 (53.0) | 1223/2097 (58.3) | 1222/2130 (57.4) | 1083/2119 (51.1) |  |
|  | >35 000 | 234/2066 (11.3) | 546/2097 (26.0) | 724/2130 (34.0) | 938/2119 (44.3) |  |
|  | Smoking |  |  |  |  |  |
|  | Never | 860/2217 (38.8) | 985/2219 (44.4) | 990/2219 (44.6) | 1027/2221 (46.2) | <0.001 |
|  | Former | 833/2217 (37.6) | 807/2219 (36.4) | 840/2219 (37.9) | 849/2221 (38.2) |  |
|  | Current | 524/2217 (23.6) | 427/2219 (19.2) | 389/2219 (17.5) | 345/2221 (15.5) |  |
|  | Drinking |  |  |  |  |  |
|  | Never | 672/2218 (30.3) | 539/2217 (24.3) | 421/2219 (19.0) | 359/2221 (16.2) | <0.001 |
|  | Former | 596/2218 (26.9) | 403/2217 (18.2) | 332/2219 (15.0) | 258/2221 (11.6) |  |
|  | Current | 950/2218 (42.8) | 1275/2217 (57.5) | 1466/2219 (66.1) | 1604/2221 (72.2) |  |
| **Physiological and Lab Variables** | | |  |  |  |  |
|  | Body mass index, kg/m^2^ | 28.7 ± 5.40 | 27.9 ± 5.22 | 27.4 ± 4.92 | 26.9 ± 5.06 | <0.001 |
|  | SBP, mmHg | 123.2 ± 17.9 | 119.4 ± 17.2 | 118.6 ± 16.5 | 117.7 ± 16.5 | <0.001 |
|  | DBP, mmHg | 72.9 ± 10.2 | 71.5 ± 9.86 | 71.1 ± 9.49 | 71.4 ± 9.60 | <0.001 |
|  | Heart rate, /min | 65.4 ± 10.6 | 65.6 ± 9.77 | 65.1 ± 9.51 | 65.1 ± 9.33 | 0.357 |
|  | Total cholesterol, mg/dl | 5.41 ± 1.01 | 5.39 ± 0.98 | 5.41 ± 0.97 | 5.41 ± 0.95 | 0.929 |
|  | HDL, mg/dl | 1.26 ± 0.43 | 1.27 ± 0.41 | 1.32 ± 0.43 | 1.39 ± 0.45 | <0.001 |
|  | LDL, mg/dl | 3.48 ± 0.95 | 3.43 ± 0.93 | 3.42 ± 0.90 | 3.37 ± 0.91 | 0.001 |
|  | Triglycerides, mg/dl | 1.49 ± 0.91 | 1.52 ± 0.89 | 1.48 ± 0.91 | 1.42 ± 0.81 | 0.003 |
|  | Creatinine, mg/ml | 1.16 ± 0.20 | 1.13 ± 0.18 | 1.12 ± 0.19 | 1.10 ± 0.17 | <0.001 |
|  | Blood glucose, mmol/l | 6.49 ± 2.51 | 6.07 ± 1.74 | 5.95 ± 1.59 | 5.81 ± 1.38 | <0.001 |
| **Chronic Medical Conditions** | | |  |  |  |  |
|  | Hypertension | 765/2212 (34.6) | 533/2217 (24.0) | 488/2215 (22.0) | 410/2317 (18.5) | <0.001 |
|  | Diabetes mellitus | 382/2202 (17.3) | 259/2216 (11.7) | 193/2213 (8.72) | 146/2217 (6.59) | <0.001 |
|  | COPD | 466/2179 (21.4) | 453/2180 (20.8) | 421/2196 (19.2) | 381/2198 (17.3) | 0.003 |

Values are expressed as n/N (%), mean ± SD, and median (25th, 75th). Global cognition z-score was calculated by computing the mean from the z score versions of the DSST, WFT, and DWRT administered during the visit 2. SBP, systolic blood pressure; DBP, diastolic blood pressure; HDL, high density lipoprotein; LDL, Low density lipoprotein; COPD, chronic obstructive pulmonary disease; DSST, Digit Symbol Substitution Test; WFT, Word Fluency Test; DWRT, Delayed Word Recall Test.

Supplementary Table 2. Adjusted ORs (95% CI) for the association of baseline (1991-1993) cognition function with incident elevated hs-cTnT and NT-proBNP.

| Variable | Quartile | Incident elevated hs-CTnT  (≥14 ng/L) | |  | Incident elevated NT-proBNP (≥300 pg/mL) | |
| --- | --- | --- | --- | --- | --- | --- |
|  |  | OR (95% CI) | P-trend |  | OR (95% CI) | P-trend |
| DWRT score |  |  | 0.011 |  |  | 0.786 |
|  | Q1 | 1.584 (1.049-2.391) | 0.029 |  | 1.011 (0.688-1.486) | 0.956 |
|  | Q2 | 1.159 (0.751-1.789) | 0.504 |  | 0.981 (0.658-1.463) | 0.925 |
|  | Q3 | 1.194 (0.758-1.879) | 0.445 |  | 1.135 (0.758-1.700) | 0.538 |
|  | Q4 | Reference | - |  | Reference | - |
| DSST score |  |  | 0.013 |  |  | <0.001 |
|  | Q1 | 1.344 (0.961-1.880) | 0.084 |  | 3.405 (1.617-6.075) | <0.001 |
|  | Q2 | 0.966 (0.714-1.308) | 0.824 |  | 2.706 (1.851-3.955) | <0.001 |
|  | Q3 | 0.821 (0.596-1.131) | 0.228 |  | 1.608 (1.091-2.370) | 0.016 |
|  | Q4 | Reference | - |  | Reference | - |
| WFT score |  |  | 0.630 |  |  | 0.638 |
|  | Q1 | 1.214 (0.906-1.625) | 0.194 |  | 1.205 (0.877-1.656) | 0.250 |
|  | Q2 | 1.135 (0.851-1.513) | 0.388 |  | 1.052 (0.774-1.43) | 0.745 |
|  | Q3 | 1.144 (0.852-1.535) | 0.371 |  | 1.143 (0.844-1.55) | 0.388 |
|  | Q4 | Reference | - |  | Reference | - |
| Language domain factor score | | | 0.484 |  |  | 0.576 |
|  | Q1 | 1.262 (0.939-1.694) | 0.122 |  | 1.256 (0.911-1.731) | 0.164 |
|  | Q2 | 1.179 (0.888-1.566) | 0.255 |  | 1.117 (0.825-1.513) | 0.473 |
|  | Q3 | 1.137 (0.847-1.525) | 0.393 |  | 1.143 (0.843-1.550) | 0.389 |
|  | Q4 | Reference | - |  | Reference | - |
| Memory domain factor score | | | 0.003 |  |  | 0.182 |
|  | Q1 | 1.265 (0.953-1.678) | 0.103 |  | 0.889 (0.663-1.193) | 0.434 |
|  | Q2 | 1.152 (0.873-1.519) | 0.318 |  | 0.890 (0.676-1.172) | 0.407 |
|  | Q3 | 0.697 (0.487-0.998) | 0.049 |  | 0.680 (0.482-0.960) | 0.028 |
|  | Q4 | Reference | - |  | Reference | - |
| Executive functioning domain factor score | | | 0.034 |  |  | <0.001 |
|  | Q1 | 1.269 (0.917-1.757) | 0.150 |  | 2.622 (1.799-3.823) | <0.001 |
|  | Q2 | 0.920 (0.682-1.241) | 0.585 |  | 1.619 (1.144-2.290) | 0.007 |
|  | Q3 | 0.838 (0.617-1.138) | 0.258 |  | 1.837 (1.320-2.556) | <0.001 |
|  | Q4 | Reference | - |  | Reference | - |

Multivariate Logistic regression analysis between cognition function and incident elevated cardiac biomarkers adjusted by age, sex, center-race, education (<high school, high school, or >high school), annual household income (<16 000, 16 000 to 25 000, 25 000 to 35 000, 35 000 to 50 000, or >50 000), smoking (never, former, current), drinking (never, former, current), body mass index, systolic blood pressure, heart rate, total cholesterol, triglycerides, high density lipoprotein, hypertension, diabetes, and chronic obstructive pulmonary disease. hs-CTnT, high-sensitive cardiac troponin T; NT-proBNP, N-terminal pro-B-type natriuretic peptide. OR, odds ratio; CI, confidence interval.
